# Neurobiological reduction: From cellular explanations of behavior to interventions

**DOI:** 10.3389/fpsyg.2022.987101

**Published:** 2022-12-22

**Authors:** David Parker

**Affiliations:** Department of Physiology, Development and Neuroscience, University of Cambridge, Cambridge, United Kingdom

**Keywords:** reductionism, psychiatry, neuroeducation, cognitive enhancement, volume transmission, ephapse, neuron doctrine

## Abstract

Scientific reductionism, the view that higher level functions can be explained by properties at some lower-level or levels, has been an assumption of nervous system analyses since the acceptance of the neuron doctrine in the late 19th century, and became a dominant experimental approach with the development of intracellular recording techniques in the mid-20th century. Subsequent refinements of electrophysiological approaches and the continual development of molecular and genetic techniques have promoted a focus on molecular and cellular mechanisms in experimental analyses and explanations of sensory, motor, and cognitive functions. Reductionist assumptions have also influenced our views of the etiology and treatment of psychopathologies, and have more recently led to claims that we can, or even should, pharmacologically enhance the normal brain. Reductionism remains an area of active debate in the philosophy of science. In neuroscience and psychology, the debate typically focuses on the mind-brain question and the mechanisms of cognition, and how or if they can be explained in neurobiological terms. However, these debates are affected by the complexity of the phenomena being considered and the difficulty of obtaining the necessary neurobiological detail. We can instead ask whether features identified in neurobiological analyses of simpler aspects in simpler nervous systems support current molecular and cellular approaches to explaining systems or behaviors. While my view is that they do not, this does not invite the opposing view prevalent in dichotomous thinking that molecular and cellular detail is irrelevant and we should focus on computations or representations. We instead need to consider how to address the long-standing dilemma of how a nervous system that ostensibly functions through discrete cell to cell communication can generate population effects across multiple spatial and temporal scales to generate behavior.

## Introduction

There is extensive debate on reductionism in the philosophy of science (Van Riel and Van Gulick, 2019), and in psychology and neuroscience (Selverston, 1980; Barlow, 1990; Gold and Stoljar, 1999; Endicott, 2001; Bickle, 2003; Bechtel, 2007; Craver, 2007; Parker, 2010; Krakauer et al., 2017). These debates consider whether one field can be eliminated by reducing it to another, and if and how component properties relate to mechanistic explanations (even the definition of mechanism is debated; see Silberstein and Chemero, 2013). These debates have continued for decades and show no sign of ending (Ingo and Love, 2022), which questions whether definitive answers are likely. Resistance from fields to being eliminated by those below is to be expected: psychology resists the claim it is a placeholder science that will be eliminated once the physiology of the brain is understood, and physiology the claim that it can be reduced to molecular biology (Noble and Boyd, 1993). There may be some professional defense in this resistance, but it is right to question what a reductionist approach can offer.

Reductionism is not a unitary phenomenon (Ingo and Love, 2022). The biologist Ernst Mayr defined three types (Mayr, 1988): constitutive reduction (functions reflect their underlying parts and their properties); explanatory reduction (mechanisms can be explained from their constitutive details); and intertheoretical reduction (a theory can be reduced to another, more inclusive theory, e.g., psychology to neurophysiology, and neurophysiology to molecular biology; Noble and Boyd, 1993; Bickle, 2003). Mayr considered constitutive reduction the simplest and least controversial, while explanatory and intertheoretical reduction were more contentious.

While debate in the philosophy of science has traditionally focused on intertheoretical reduction, constitutive, and explanatory reductionism have come to the fore (Ingo and Love, 2022). These forms of reduction have been related to a mechanical or “machine model” where outputs are generated by parts that perform specific functions. Ingo and Love (2022) wrote, “mechanisms are understood as akin (though not equivalent) to machines with interconnected, organized parts operating to produce regular or expected outcomes,” following Alberts (1998) who compared a cell to a factory where specific functions are performed sequentially along chains of protein machines (see also Reynolds, 2007). Hanahan and Weinberg (2000, p. 67) updated the machine analogy by claiming, “Two decades from now…it will be possible to lay out the complete integrated circuit of the cell…we will then be able to apply the tools of mathematical analysis to explain.” The integrated circuit analogy is notable as Jonas and Kording (2017) have shown that current reductive approaches applied to actual integrated circuits fail to explain their function. Hanahan and Weinberg’s two decades have now passed, but instead of a complete integrated circuit features have been identified that negate the integrated circuit analogy. These include a fluid cytoskeleton, “intrinsically disordered proteins,” enzymes with numerous substrates or that perform non-enzymatic functions, pleomorphic molecular assembles with “probability clouds” of interactions, and probabilistic gene expression (see Nicholson, 2019). These aspects do not negate reductionist approaches in principle but show that previous assumptions and metaphors were simplistic.

Broadly speaking, given the controversy over definitions (Ingo and Love, 2022), psychoneural, or neurobiological reduction sees psychology and behavior explained mechanistically in terms of the constituent molecules and cells, and can include some combination of constitutive, explanatory, and intertheoretical reduction. Constitutive and explanatory reduction has been dominant aspects in neurobiology for several decades (e.g., Selverston, 1980; Getting, 1989; Ito, 2006; Yuste, 2008). These analyses have led to significant insight into cellular and synaptic properties. Some have claimed causal explanations of behavior from these analyses (see Parker, 2006, 2019 for examples and critique), and where gaps in mechanistic schemes are acknowledged it is assumed that reductive approaches will ultimately be successful. For example, in reviewing the link between the long-term potentiation (LTP) of hippocampal synapses and memory, Bliss et al. (2018, p. A105) admitted “definitive proof that the mechanisms of LTP subserve learning and memory in the behaving animal is still lacking,”…but they went on to say that “few neuroscientists doubt that such proof will eventually be forthcoming.”

To consider constitutive and explanatory reduction in neurobiology, I will start with the basic issue of experimentally identifying component neurons, and then consider how the organization of neurons in neural circuits affects our ability to offer reductive or mechanistic explanations. I will finish by considering claims that our mechanistic knowledge of the nervous system obtained in reductionist analyses is sufficient to be translated into practical uses. These claims extend beyond interventions in traditional areas like neurology and psychopathology to include aspects of normal cognition and behavior, with some claiming that not only can we safely and effectively intervene in the normal brain, but also that we should.

## The identification of components and their roles

The reductionist belief that molecular and cellular properties underlie cognition and behavior has been called the neuron doctrine (e.g., Barlow, 1972; Gold and Stoljar, 1999). This doctrine takes different forms with different implications: the trivial neuron doctrine sees psychological explanations remaining autonomous despite being implemented by neuronal properties, while the radical doctrine sees psychological aspects explained by neuronal properties (see Gold and Stoljar, 1999). The term neuron doctrine originated at the end of the 19th century with acceptance that the brain is made of discrete cells rather than being a continuous reticulum (Shepherd, 1991). This became an experimental focus in the 1950s with the development of techniques for intracellular recordings from single cells (Bickle and Parker, 2022) and is referenced in the terms like command neuron, place cell, grandmother cell, gnostic unit, and feature detector (e.g., Barlow, 1972; Hubel, 1974; Edelman, 1989; Zeki, 1993; Crick, 1994; Changeux, 1997; Kandel, 1998). Gold and Stoljar (1999, p. 2–3) quote several philosophers and neuroscientists who claim that nervous system functions can be explained from cellular components. For example, Churchland and Sejnowski claimed “it is highly improbable that emergent properties cannot be explained by low-level properties”; Semir Zeki wrote that “It is only through a knowledge of neurobiology that philosophers of the future can hope to make any substantial contribution to understanding the mind”; Gerald Edelman said a theory of the brain needs “a description based on the neuronal and phenotypic organization…formulated solely in terms of physical and chemical mechanisms giving rise to that organization”; and Francis Crick that “A person’s mental activities are entirely due to the behavior of nerve cells, glial cells, and the atoms, ions, and molecules that make them up.” Crick made the definitive reductionist statement, “All approaches at a higher level are suspect until confirmed at the molecular level” (Crick, 1988, p. 61). These views suggest that once all the relevant component molecules, cells, and interactions have been characterized we will understand function, a neuroscience version of Laplace’s demon (Laplace, 1902).

The experimental criteria claimed for a reductive explanation of behavior in neurobiology have been outlined several times (they have been repeated, albeit using different terms, by philosophers of neuroscience in their discussions of various reductive approaches; Ingo and Love, 2022). Neurobiological criteria reflect the need to identify the component neurons involved in a behavior, their direct synaptic connections, and the functional properties of specific classes of neurons and synapses; this information is ultimately integrated to try to provide a unified explanation of the behavior (e.g., Bullock, 1976; Selverston, 1980; Getting, 1989; Yuste, 2008; Braganza and Beck, 2018). These criteria have been applied to experimental analyses in invertebrate and vertebrate nervous systems, traditionally using electrophysiological and anatomical techniques and now also using various imaging, molecular genetics, and optogenetic approaches. They remain a major focus of neuroscience research and tool development. Thus, the US BRAIN initiative explicitly aims to develop new tools for reductionist analyses, stating “By accelerating the development and application of innovative technologies, researchers will be able to produce a revolutionary new dynamic picture of the brain that, for the first time, shows how individual cells and complex neural circuits interact in both time and space.”^1^ These analyses and their assumptions have also influenced our views of psychopathology, with aberrant functions being considered to reflect genes, neurotransmitters, and other signaling molecules that can be targeted in interventions: thus, the BRAIN initiative promises “new ways to treat, cure, and even prevent brain disorders.”^1^

Much of the debate around reductionist approaches assume that we can obtain or have obtained the necessary component detail, debate focusing on what this data can explain. But even the correct identification of component cells, a crucial step in a mechanistic explanation, is far from trivial (Selverston, 1980; Parker, 2006). Components, either molecules, cells, or brain regions, underlying different functions have been identified using the criteria of necessity and sufficiency. This traditionally used lesions, electrical stimulation, or pharmacological activation or inhibition, and now includes molecular genetic and optogenetic loss and gain of function approaches, with outputs assessed from behavior or by imaging or recording from neurons or brain regions. A necessary condition must be present for an effect to occur, shown by the correlated activity of a component with an effect and the absence of the effect when the component is silenced; sufficiency is shown when the activation of a component can evoke the effect. While these criteria have been used experimentally for many years, there are long-standing issues with them. These have been discussed in the context of simpler nervous systems where direct links, or the lack of them, are easier to examine (e.g., see debate over the command neuron concept in Kupfermann and Weiss, 1978).

**Figure 1:**
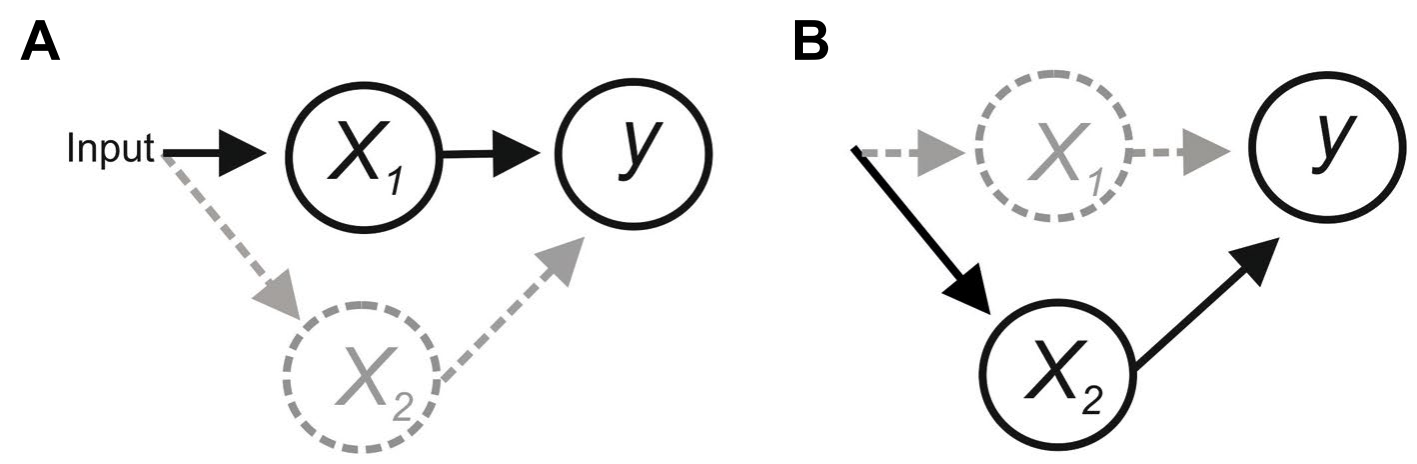
**(A)** Activation of *x_1_* is necessary and sufficient for activating *y*. But if an alternative pathway allowed *x_2_* to activate *y* then *x_1_* would be sufficient but not necessary **(B)**.

Consider the scheme in Figure 1. In (*a*), *x_1_* is the only functional connection onto *y*, and activity of *y* is evoked/abolished when *x_1_* is activated/inactivated, suggesting that *x_1_* is necessary and sufficient for *y*. However, degeneracy (i.e., different components can perform the same function; Tononi et al., 1999) or compensatory plasticity that can rapidly adapt to a perturbation to maintain function (Davis and Bezprozvanny, 2001; Frank et al., 2006) could allow *x_2_* to substitute for *x_1_*, making *x_1_* sufficient but not necessary for evoking *y* (b).

**Figure 2:**
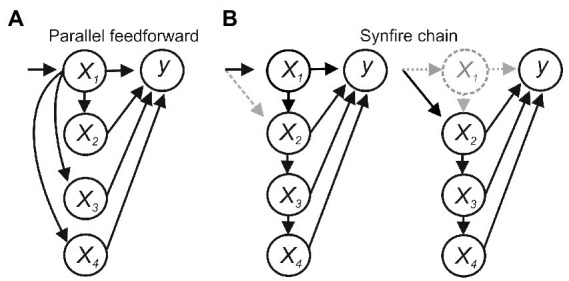
**(A)** Parallel feedforward connections. *x_1_* would be necessary for activating *y* but not sufficient if the summed input from *x_1_*– *x_4_* was needed. **(B)** A synfire chain. *x_1_* would not be sufficient if the summed input from *x_1_*– *x_4_* was needed and would not be necessary if degeneracy or compensatory plasticity allowed *x_2_*–*x_4_* to activate *y*.

Feedforward connections between *x_1-4_* introduce additional issues. In Figure 2A, *x_1_* sends parallel feedforward projections to *x_2_–x_4_*, which sum to activate *y*. Activating *x_1_* will evoke *y*, and blocking *x_1_* will block *y*, suggesting *x_1_* is necessary and sufficient for *y*, but *x_1_* would not be sufficient if the summed input from *x_1_–x_4_* was needed to activate *y*. In a synfire-like chain (Figure 2B), activating *x_1_* will evoke *y* and inhibiting *x_1_* will block *y*, again suggesting *x_1_* is necessary and sufficient. But *x_1_* may again not be sufficient if the summed input from *x_1_* to *x_4_* was needed, and in this case would also not be necessary if degeneracy or compensatory effects allowed *x_2_* to recruit *x_3_–x_4_* to evoke *y*.

**Figure 3:**
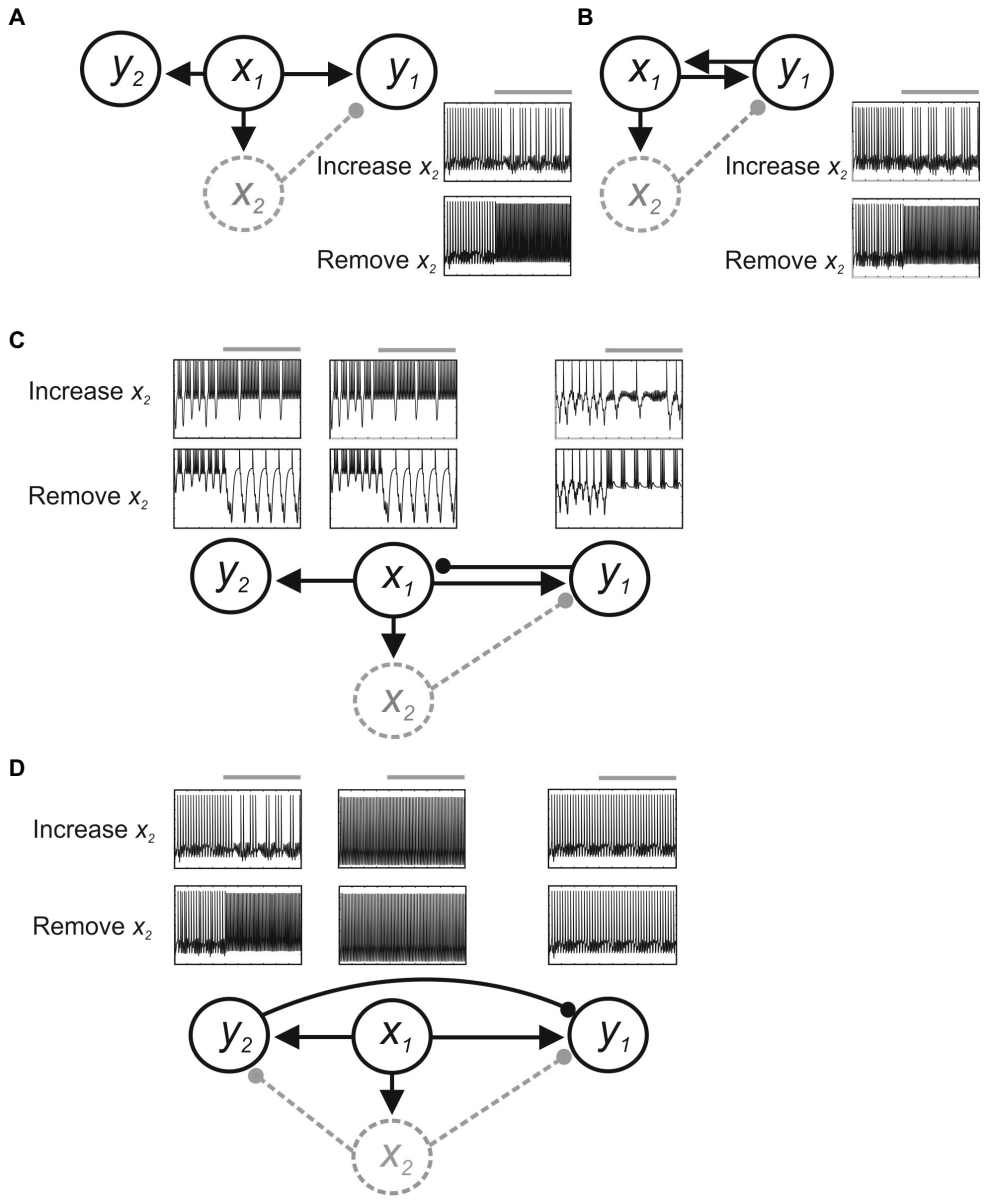
Feedback effects in a simple model. Neurons are modeled using Hodgkin-Huxley kinetics, and inhibitory (filled circle) and excitatory synapses (arrow) are modeled using alpha functions. The circuit is driven by a constant excitatory input to *x_1_*. **(A)** With only feedforward connections, positive and negative manipulations of *x_2_* decrease or increase *y_1_* activity. **(B)** Feedback excitation from *y_1_* to *x_1_* causes oscillation of *y_1_* activity when *x_2_* inhibition is increased. **(C)** Feedback inhibition from *y_1_* to *x_1_* can also evoke oscillation in *y_1_*, *x_1_*, and in *y_2_* as a result of diaschisis. **(D)** With inhibition from *y_2_* to *y_1_* positive and negative manipulations of *x_2_* may cause no change in *y_1_*.

Feedback connections add further issues. Figure 3 shows effects in a simple computer simulation (Jia and Parker, 2016). Here, *x_1_* sends parallel excitatory inputs to output neurons *y_1_* and *y_2_*, and to interneuron *x_2_*. Assume *y_1_* generates the output underlying a behavior we are investigating, and we positively and negatively manipulate *x_2_* to test the hypothesis that it inhibits *y_1_*. Without feedback connections (Figure 3A), activating or inactivating *x_2_* reduces or increases *y_1_* activity, respectively, consistent with the hypothesized inhibitory role of *x_2_* (albeit subject to the provisos of degeneracy and compensation outlined above). However, with feedback excitation from *y_1_* to *x_1_* (Figure 3B), removing *x_2_* will increase *y_1_* activity, as hypothesized, but increasing *x_2_* activity will cause oscillation rather than inhibition because (1) increased inhibition from *x_2_* reduces *y_1_* activity; which (2) reduces feedback excitation of *x_1_*; which (3) reduces *x_2_* activation and disinhibits *y_1_*; (4) this increases *y_1_* activity and thus feedback excitation of *x_1_* and *x_2_* activity to reduce *y_1_* activity; and (5): the cycle repeating to cause oscillation. With feedback inhibition from *y_1_* to *x_1_* (Figure 3C), removing *x_2_* will increase *y_1_* activity, but as this inhibits *x_1_* the excitatory drive to *y_1_* and *y_1_* feedback inhibition of *x_1_* will be reduced, again causing oscillation in *y_1_* as *x_1_* activity increases and decreases. Finally, as *x_1_* connects to *y_2_*, any changes in *x_1_* will alter *y_2_*, even though neither *x_1_* nor *y_2_* is directly affected by *x_2_* and *y_2_* has no role in the function. This is an example of “diaschisis” (Carrera and Tononi, 2014; Otchy et al., 2015), a neurological term seemingly less appreciated experimentally that means “shocked throughout” to represent the widespread system changes evoked by even very precise manipulations of system components.

Changes in *y_1_* thus occur that are not predicted from manipulation of *x_2_*. An added issue is that if *x_2_* directly affects *y_2_* then diaschisis could also result in *y_1_* activity being unaffected despite widespread changes in functionally relevant system components. In Figure 3D, *x_2_* inhibits both *y_1_* and *y_2_*, and *y_2_* provides feedforward inhibition of *y_1._* Removing *x_2_* inhibition will increase *y_1_* activity, but it will also increase *y_2_* activity through disinhibition. This could evoke inhibition of *y_1_* that leaves *y_1_* activity unchanged, a negative result that could erroneously suggest no influence of *x_2_* in the circuit.

Degeneracy, compensatory plasticity, diaschisis, and feedforward and feedback connections, all established aspects of nervous systems, can thus complicate interpretations of even totally precise and controlled manipulations of component parts (note that even the most advanced molecular techniques are promiscuous and can affect more than the intended target (Newton et al., 2019), negating the “surgical” analogy that they allow molecular dissection of circuits; Kiehn and Kullander, 2004). Misinterpretations can lead to the erroneous inclusion or omission of components in mechanistic schemes, with obvious consequences to claimed explanations, understanding, and interventions. We could claim that with sufficient (Laplacian?) knowledge these issues would be recognized and correct explanations would be provided, but while easily seen in these cartoon examples could we readily identify these features in more complex circuits? The practical and conceptual challenges of reductive approaches that link component parts to functions have been highlighted several times in invertebrate and lower vertebrate nervous systems containing relatively few, often large and uniquely identifiable cells (Kupfermann and Weiss, 1978; Selverston, 1980, 2010; Getting, 1989; Parker, 2010). These features should make the linking of components to functions easier than analyses of cognition in mammals, but even in these systems (tellingly referred to as “simpler” rather than “simple”) errors have been made and gaps remain in explanatory schemes after decades of analysis (e.g., Selverston, 1980, 2010; Parker, 2006).

## Relational aspects in reductionist schemes

Identifying components is only the first step in a mechanistic explanation. Neurobiological criteria for reductive explanations highlight the requirement of knowing how component cells are synaptically connected in a system organization or architecture, and the functional properties of the cells and synapses that allow them to perform their functions. Crick (1994, p. 3) claims that “your joys and your sorrows, your memories and ambitions, your sense of personal identify, and freewill, are in fact *no more than* [my italic] the behavior of a vast assembly of nerve cells,” downplays the importance of the assembly. For example, sodium channel function does not simply reflect a vast assembly of molecules but requires cooperativity between appropriately arranged parts (e.g., voltage-sensitive S4 regions; Marban et al., 1998). This reflects the folding of the channel polypeptide chain, which depends on the amino acid sequence, interactions between amino acids, extrinsic factors (“chaperone” proteins), and the physico-chemical properties of the environment (hydrophobic amino acids orientate internally), with channel function ultimately reflecting the properties of the whole cell (e.g., voltage and electrochemical gradients). The functions that Crick poetically refers to reflect specific populations of neurons that make specific synaptic connections and have neuron and synapse-specific functional properties. To claim a reductionist decomposition of a system of *n* cells requires characterizing some *>n* synaptic connections and some *> > n* cellular and synaptic properties. This was highlighted by Selverston (1980), Getting (1989), and later by Koch (2012) who (albeit in a straw man assumption of all-to-all connectivity) used Bell’s number to calculate that it would take 2,000 years to completely characterize the direct connectivity of a system of 1,000 fully interacting components.

This demonstrates why reductionist analyses even in nervous systems that contain only 100s or even 10s of neurons do not examine every component and interaction. Analyses instead define neurons as belonging to populations, either by the region they are in or some cellular marker (e.g., GAD2 as a claimed marker of inhibitory; i.e., GABAergic, neurons; Quina et al., 2020), or focus on more tractable larger cells like motor neurons, hippocampal and cortical pyramidal neurons, or cerebellar Purkinje cells instead of the smaller and often more numerous interneurons. Connectivity is often examined using indirect methods, extracellular stimulation of presynaptic neurons, and statistical models of connectivity (Horwitz, 2003), using criteria that can fail to correctly identify direct connections (Berry and Pentreath, 1976; Parker, 2010). Grouping neurons and synapses into populations characterized by mean values is a necessary and acceptable approach providing that we appreciate that this may remove functionally-relevant variability (Parra et al., 1998; Aradi and Soltesz, 2002; Golowasch et al., 2002; Soltesz, 2006; Parker and Srivastava, 2013).

While these approaches are necessary, they leave mechanistic descriptions lacking detail on identified component neurons and their specific synaptic interactions, features highlighted as necessary criteria for reductive analyses in neurobiology (Bullock, 1976; Selverston, 1980; Getting, 1989). We can of course debate whether this level of detail is needed (see the commentaries in Selverston, 1980 for an early debate; Parker and Srivastava, 2013), but we cannot simply appeal to experimental convenience or tractability in the components we include (i.e., we cannot just ignore aspects that we cannot currently examine, but need to highlight the absence of potentially important details). Approaches may be field-dependent: neurophysiological analyses will typically attempt to identify specific neurons and interactions but may pay little attention to molecular aspects or behavior (Krakauer et al., 2017), while very detailed molecular analyses and manipulations may be examined at the neurophysiological only on unidentified or crudely characterized neurons or those that are experimentally tractable. A causal mechanism would seem to require that we know how the simultaneous integrated activity of specific types of cell, their properties, and their specific interactions in circuits generate a behavior, otherwise we can only correlate some molecular or cellular property to a behavior (a correlation is not necessarily uninformative).

The lack of relevant detail does affect explanations. In my field, the claimed experimentally characterized the lamprey spinal cord locomotor network in reality uses several assumptions and extrapolations to cover missing details and uncertainties over components, their connectivity, and functional properties (Parker, 2006, 2010). Likewise, the analysis of the ~200 interneurons involved in the *Aplysia* gill-withdrawal reflex was described as “forbidding in its complexity” by Hawkins et al. (1981), most subsequent work focusing on the experimentally tractable sensory neurons, an analytically convenient approach that fails to provide the claimed, and widely accepted, causal account of the behavior because it ignores known changes in motor neurons and interneurons (see Glanzman, 2010 and Trudeau and Castellucci, 1993; see Parker, 2019 for review).

New techniques promise to overcome analytical limitations. For example, the BRAIN initiative claims understanding will follow from recording “from ever more cells over larger brain regions” (Mott et al., 2018, p. 3); connectomic analyses claim that functional explanations will follow from more detailed brain mircoanatomy (Morgan and Lichtman, 2013; Schroter et al., 2017); and the originator of the Human Brain Project, Henry Markram, claimed that a more detailed cortical column model will cause a Copernican revolution in neuroscience (see Lehrer, 2008). These claims reflect an illusion of depth (Ylikoski, 2009). An explanation, let alone a Copernican revolution, is not a reflection of how many components we monitor or manipulate but of knowing what this data means in an explanatory scheme. An example is provided by Greenberg and Manor (2005) who modeled the pyloric network of the crustacean stomatogastric ganglion, a system containing relatively very few neurons that is arguably the best understood neural circuit. Greenberg and Manor went beyond modeling the usual three neuronal groups to include five types of circuit neurons and their connections. They showed that an interaction resulting from the combination of an A-type potassium current and short-term synaptic depression was needed to generate the normal pyloric rhythm, but the complexity of the model, which consisted of almost 50 coupled differential equations, prevented them from explaining the underlying mechanism. As a result, they reverted to the use of a simpler model, stating “The reduced model emphasizes a result that is difficult to discern in the detailed model because of its complexity” (Greenberg and Manor, 2005, p. 676).

Simon (1962) suggested that mechanistic explanations are in principle possible irrespective of the number of components and interactions if systems are decomposable or nearly decomposable, namely have a fixed hierarchy of components where intracomponent interactions are strong but intercomponent interactions are relatively weak (but non-negligible), and each component processes the input it receives from the component above it in the hierarchy: this makes the behavior of each component approximately independent of the behavior of the others. The decomposability of nervous systems was examined by Bassett et al. (2010) using the connectome of the *C. elegans* nervous system and human brain fMRI data. They claimed that both showed “some” degree of hierarchical organization, and cited Simon in claiming that they are thus nearly decomposable. But Simon did not say that even fully hierarchical systems are nearly decomposable, just that “some kinds of hierarchical systems can be approximated successfully as nearly decomposable systems” (Simon, 1962, p. 474).

Being decomposable or nearly decomposable is a core assumption of reductionist approaches. For example, when we manipulate a system component we assume that the resulting effect reflects the function of that component. We should consider the validity of our assumptions, and an obvious consideration is whether nervous systems are decomposable and whether we can directly link a manipulation to an observed effect. A system is minimally or non-decomposable if interactions between components are many or strong and the function of a component reflects not only its intrinsic properties but also its relationships with other components. This seems to better describe nervous systems, which consist of multiple parallel feedforward, lateral, and feedback pathways (Sporns, 2011; Pessoa, 2014). For example, cortical areas, including primary sensory regions, receive parallel convergent inputs from various sources that make the regions multifunctional, while feedback connections from these regions can influence the nature of the incoming inputs that they process (Anderson, 2010; Schroeder and Foxe, 2005). Conversely, specific functions can be performed by multiple regions. A classic example of this is Lashley’s equipotentiality hypothesis that suggested that memory is stored diffusely in multiple cortical areas (Lashley, 1929). Re-analysis of Lashley’s data resulted in some quantitative modifications but his general conclusions have held (see Thomas, 1971), and despite the localization of the molecular and cellular mechanisms of memory being a major focus of neuroscience research over the last 4 decades (Bliss et al., 2018), Josselyn et al. (2015, p. 521) wrote that the failure to localize the engram reflects the “widely distributed and dynamic nature of memory representations in the brain.”

These distributed and multifunctional effects necessarily complicate the mapping of specific functions to specific regions or components. This was highlighted by the neurophysiologist Charles Sherrington who called reflexes a “convenient fiction…a simple reflex is probably a purely abstract conception, because all parts of the nervous system are connected together and no part of it is probably ever capable of reaction without affecting and being affected by various other parts” (Sherrington, 1906, p. 8). McCulloch (1945) highlighted that parallel feedback and feedforward connections in what he called heterodromic systems were necessary to co-ordinate behavioral responses, and claimed that even simple heterarchical systems are unpredictable because their connectivity allows component relationships, their independence, and their importance and ordering can change. This view has supported been supported experimentally by the heterarchical organization of spinal cord sensorimotor systems (see Cohen, 1992) and by the switching of molecular and cellular components between functions (e.g., Meyrand et al., 1991; Daaka et al., 1997; Fahoum and Blitz, 2021). These aspects do not deny hierarchical processing occurs, but there is not a fixed hierarchy as a components role and position can change depending on context. A heterarchical organization does not prevent explanations of non-decomposable systems but does require that explanations consider variable relational aspects rather than seeking 1-to-1 links. This was highlighted by Rashevsky (1954) who referred to “metric biology” for analyses of system components, and “relational biology” for aspects dependent on system organization. Specific analyses are needed because new properties can appear at different levels (Anderson, 1972).

Relational aspects oppose substantivalist views that see functions represented in components, expressed in references to memory molecules, inhibitory or excitatory neurotransmitters or neurons, and mood or reward neurotransmitters. Neurons and neurotransmitters are not intrinsically inhibitory or excitatory, as can often be claimed (e.g., Eckstein et al., 2020). Inhibition and excitation as well as functions like mood (serotonin), reward (dopamine), and pain (substance P) are not intrinsic to a neuron or neurotransmitter but depend on the transmitter receptor activated, the cells the receptors are in, and the circuits/regions where the cells are located (dopamine is also involved in retinal processing (Korshunov et al., 2020), 5-HT in motor control (Jacobs and Fornal, 1993), and substance P in breathing (Pilowsky, 2014)). Consider the neurotransmitter GABA. Identification of GAD2, the enzyme that synthesizes GABA is often used to identify “inhibitory” neurons (seer Quina et al., 2020). But GABA itself is not inhibitory: ionotropic GABA_A_ receptors are permeable to Cl^−^, but whether this evokes inhibition or excitation depends on whether chloride enters or leaves to hyperpolarize or depolarize the cell (shunting inhibition can occur if there is no net movement of Cl^−^). This depends on the equilibrium potential for chloride, which in turn depends on the activity of Cl-pumps that determine the intracellular Cl-levels and the membrane potential of the cell, all of which will simultaneously change as the neuron and receptor are activated. Even if a GABAergic neuron was known to hyperpolarize and inhibit a postsynaptic cell this may still not describe its functional effect as inhibition of other inhibitory neurons (disinhibition) will evoke excitation.

Relational aspects are illustrated by homeostatic plasticity where parameter values vary as a function of the variability of other components to maintain an output. In single cells this can reflect variations in different classes of ion channels or synaptic inputs to maintain a certain level of cellular excitability (Turrigiano et al., 1998; Swensen and Bean, 2005), while in neural circuits variability in neuronal and synaptic properties can maintain a particular circuit output providing that the ratios between the different functional components are in appropriate balance (see Prinz, 2010). Examples of the latter include the 4,000,000 combinations of eight types of ion channels and seven types of synapse that could generate the modeled output of a three neuron stomatogastric ganglion circuit (Prinz et al., 2004); compensations in basal ganglia circuitry that delay Parkinson’s disease symptoms until 80% of the dopaminergic neurons in the substantia nigra have degenerated (Bezard et al., 2004), and in functional recovery from spinal cord lesions where locomotor behavior matching that in unlesioned animals according to various behavioral measures can be generated using spinal cord systems with markedly different anatomical and functional properties (Davis et al., 1993; Edgerton et al., 2001; Parker, 2017).

These variable relational effects show that multiple neurophysiological states (N_1_ v N_2_…N_n_) can realize a single behavior or cognitive process (P_1_ ↔ N_1_ v N_2_…N_n_). This could be considered a neurobiological example of multiple realisability, although this is a contentious issue (see Aizawa and Gillett, 2009). But the evidence above suggests that nervous system outputs are linked to multiple, not single neurophysiological states, even when the cellular properties and the output are both measured in comparable detail (Aizawa and Gillett, 2009). Multiple realization is claimed to prevent reductive explanations (see Aizawa and Gillett, 2009 for discussion) but this seems not to be the case in the examples above. But it does require that variable relational effects between components are known, and that we know when and why one or other particular neurophysiological state is used.

Relational aspects are not confined to interactions within the nervous system but also reflect interactions of the nervous system with the body (e.g., proprioceptive, neural-immune, and gut-brain interactions) acting in the environment (see Dreyfus, 2012). This has been called embodied cognition (Shapro and Spaulding, 2021) and has been inspired by ecological psychology and neuroethological analyses (Chiel and Beer, 1997). These effects move away from the view that the nervous system sequentially processes inputs to plan and generate outputs to one where adaptive behavior results from the continuous two-way relationships between the nervous system, the body, and the environment. These are referred to as levels, but while this may provide a simplifying concept for organizing data and analyses it also gives the erroneous impression of effects working up or down through separate stages when all effects are occurring simultaneously (Noble, 2012). There are many examples of behavioral and environmental influences on brain function: adult neurogenesis is enhanced in enriched environments (Altman, 2011); the availability of receptive females influences male primate and human testosterone levels and sexual behavior (Anonymous, 1970); amphetamine effects on primate behavior reflect position in the social hierarchy (see Cacioppo and Berntson, 1992); and hyperactivity and low blood levels of serotonin, a correlation that could have led to a claimed causal link, were both normalized in children when they were hospitalized (Coleman (1971).

It could be argued that as embodied effects are represented in the molecular transduction of sensory neurons and through various sub-cortical and cortical sensory processing stages, they can be incorporated into the neurobiological explanation. But this would require consideration of heterarchical processing that is continuously altered by internal and external relationships between ongoing functions and behaviors. As McCulloch (1945) suggested, relational contexts mean that even though an input is represented by a pattern of sensory activity, this activity won’t necessarily predict the resulting effect. The placebo effect in pain perception would be an example, where an external context, expectation of analgesia, leads to an alteration in nervous system processing through activation of endogenous opioid systems that alters the perception of the sensory input and the resulting behavior (Benedetti, 2007).

Noble (2012) uses the heart to illustrate these relational effects. Even though genes for various cardiac ion channels specify heart cells over other cell types, the heart rhythm is not determined by these genes but by the component ion channels, cellular properties (electrochemical potentials) that affect ion channel activity, gross heart structure, the ongoing heart rhythm, and internal and the external environmental factors that influence it. Relational aspects were demonstrated when computing advances in the 1990s allowed the detailed information obtained on cardiac ion channels to be incorporated into multicellular models of the sino-atrial node pacemaker. In the model, cells at the edge of the node depolarized first and activity spread inwards, but in the heart the activity originates near the center of the node and spreads outwards. When the sino-atrial node was dissected from the atrium it behaved like the computer model, normal activity thus reflecting relational influences arising from the organization of the heart (see Noble et al., 2019).

Simon (1969, p. 52) summarized these wider relational effects, “A man [*sic*], viewed as a behaving system, is quite simple. The apparent complexity of his behavior over time is largely a reflection of the complexity of the environment in which he finds himself.” But this can be extended because environments are also continuously modified by ongoing behavior (see Chiel and Beer, 1997), a circular interaction. There is nothing mystical or metaphysical about these higher-level context-dependent effects. They can be expressed mechanistically and mathematically with the same precision as lower-level mechanisms, the latter using differential equations, and higher-level context-dependent influences by the initial and boundary conditions of these equations (Noble et al., 2019).

As mentioned above, relational effects complicate experimental approaches that attempt a 1-to-1 mapping of components to functions (e.g., reverse inferences in brain imaging studies), and the interpretation of system manipulations. Newer techniques like gene knock-outs and optogenetics claim greater precision than traditional approaches (e.g., physical lesions or pharmacological approaches), but no matter how surgical a manipulation is, relational effects mean that there will necessarily be changes in the properties of other components (i.e., diaschisis). This is of course the aim of a manipulation, to identify a component and its role from how the system changes after its manipulation. In decomposable or nearly-decomposable systems where parts are relatively independent we could relate the resulting system changes to the manipulated component, but relational effects in non-decomposable systems mean that system effect will reflect changes in more than the manipulated component (or no system effect despite key components being altered; Figure 3D) thus requiring us to consider multiple causes for a system effect. Also, while we assume that we can at least be confident that we can precisely control the component we have manipulated, feedback pathways in relational systems can affect the manipulated component and thus the system is not manipulated in the way we intended. Both of these aspects necessarily complicate attempts to localize system functions to specific component parts.

An additional aspect of relational effects is that when a system is inactive or its normal organization is disturbed (e.g., in cell cultures or tissue slices, routine experimental approaches used because they provide us with greater access and control over a system) the properties that we characterize can differ to those in the intact, active system. Claude Bernard wrote, “the phenomena of a living body are in such reciprocal harmony one with another that it seems impossible to separate any part without at once disturbing the whole organism,” quoting Georges Cuvier, “All parts of a living body are interrelated; they can act only in so far as they act all together; trying to separate one from the whole means transferring it to the realm of dead substances; it means entirely changing its essence” (see Normandin, 2007). An example of a change in essence in dissected or quiescent systems is the absence of functional properties normally established by relationships in the intact, active system. These are not components in the traditional sense that can be isolated; they do not exist in specific locations with specific values or even exist at all under some conditions. These effects include volume transmission and ephapses (Faber and Pereda, 2018; Svensson et al., 2019), both of which negate the claim that “wired” axonal and synaptic connections determine functional interactions in nervous systems (e.g., Price and Friston, 2005).

Ephaptic signals reflect changing electrical fields in the extracellular space generated by summed neuronal activity. The membrane potential (*V*_m_) is the difference between the intracellular (*V*_i_) and extracellular potential (*V*_e_), *V*_m_ = *V*_i_ − *V*_e_. Current flow to or from the local extracellular environment caused by cellular or synaptic activity generates local field potentials that change *V*_e_ and thus *V*_m_. These effects are anisotropic, the magnitude and direction of the change in *V_e_* reflecting a complex interaction of several variables including the number of active cells, the pattern of their activity, the packing and orientation of neurons and processes, the geometry of the extracellular space, and the properties of the “postephaptic” cell (e.g., location of ion channels in an ephaptic field). Ephapses provide a concrete neurobiological example of an emergent effect. While these effects can be modeled, the equations currently rely on assumptions of several unknowns. We know field effects occur, they are measured in EEGs, but are they functionally-relevant signals (e.g., Bullock, 1959) or an epiphenomenon of neural activity? The latter view has seemingly dominated with the experimental focus on single cells and synaptic connections.

To consider the implications of ephapses to reductionist explanations, assume that field effects are important. They are generated by neuronal activity and neuronal activity is altered by field effects, a circular interaction. But neuronal activity also alters the geometry of the extracellular space (Østby et al., 2009), meaning that even if all the variables listed above were characterized, they will all continually change during system activity: a change in the extracellular space will alter the magnitude and spread of field effects, which will alter neuronal and system activity, and thus alter the extracellular space… a circular interaction influencing another circular interaction.

We cannot explain these effects by describing individual cellular or system properties but must consider the relationships between local and global effects simultaneously. This was expressed in Lashley’s dilemma, “Nerve impulses are transmitted from cell to cell through definite intercellular communication. Yet all behavior seems to be determined by masses of excitation” (Lashley, 1942, p. 306). We can appreciate this from the intuitive sense of our own behaviors, which does not support a mechanistic sequence of effects passed from one element to another along axons and across synaptic connections. Take movement: robotic systems split movements into sequences of distinct parts, but in a natural movement like reaching for a cup you do not first move your shoulder, then elbow, then wrist, then fingers: movement at the beginning (shoulder) and end (hand/finger) may change, but as the shoulder moves the wrist or fingers are shaped to be in position when the hand reaches the cup. Bullock (1959, p. 999) offered a potential solution by extending the neuron doctrine in saying “perhaps much of the normal functioning is carried out without nerve impulses…by means of graded and decrementally spreading activity,” and proposed, like Sherrington had for reflexes, that circuits of wired interacting components are an “oversimplified abstraction involving a limited subset of communicated signals…in fact, there are many parallel types of signals” (Bullock, 1981, p. 281). Despite his optimism that “in the near future we will gain significant new insight” (Bullock, 1959), these ephaptic signals have received very little attention compared to single molecules, cells, and wired synaptic connections. This is starting to change as functional ephaptic effects have been shown and studied in several systems (Faber and Pereda, 2018).

Volume transmission is the diffusion of neurotransmitters through the extracellular space to affect targets distant from their release sites (μm for amines and mm for neuropeptides; Svensson et al., 2019): anatomical localization thus does not determine a transmitter’s effects (*cf* Price and Friston, 2005). Volume signaling is not simply a synaptic signal spread over a wider area but like ephaptic effects is anisotropic, the direction and extent depending on the size and charge of the transmitter, the activity-dependent geometry of the extracellular space, the presence of or efficacy of uptake or breakdown mechanisms, charges on extracellular proteins or ephaptic field potentials that attract or repel molecules, and even “tidal” effects caused by blood pulsing in arteries. Like ephapses, volume effects thus reflect changing spatial and temporal relationships between components.

Volume transmission also allows two or more transmitters released from spatially distant regions to interact (interactions can also occur locally through co-release from single synaptic terminals or vesicles; Svensson et al., 2019). Transmitter interactions are a highly conserved basic feature from invertebrate to mammalian nervous systems that can generate additive, subtractive, non-linear, or emergent effects (i.e., effects not associated with any individual transmitter; Brezina, 2010; Svensson et al., 2019). Amines and neuropeptides act on G protein-coupled receptors and intracellular pathways to modulate the functional properties of cells and synapses from seconds to hours (Svensson et al., 2019). They can thus evoke a background “modulatory tone” that allows interactions between transmitters whose release is not only spatially but also temporally divorced.

**Figure 4:**
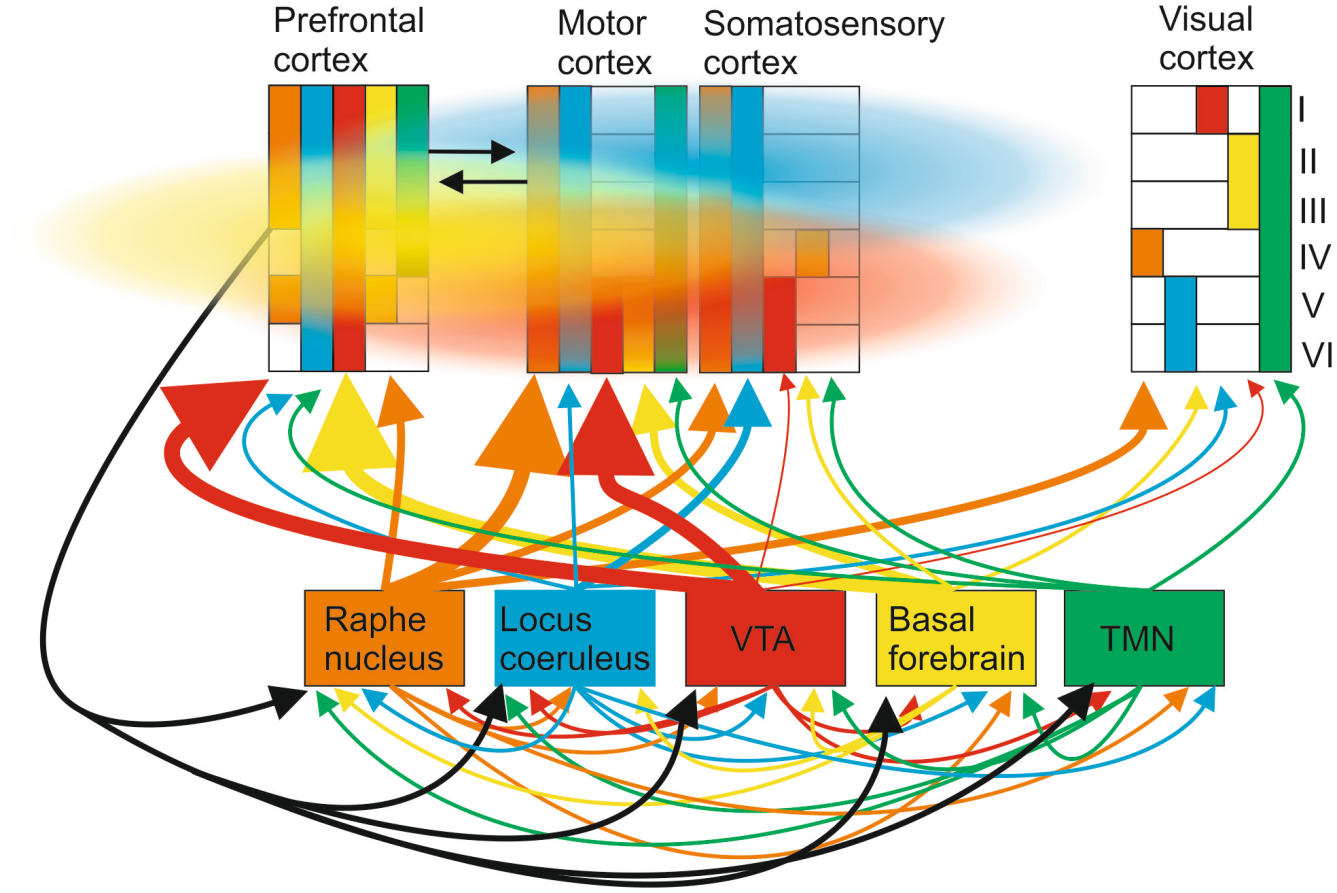
Ascending modulatory pathways make wired connections to multiple cortical areas (regional projections to different cortical areas and layers are indicated by the size of the ascending arrow and the colored blocks in the different cortical regions; layers are indicated by the roman numeral on the right). These include cholinergic inputs from the basal forebrain, noradrenergic inputs from the locus coeruleus, serotonergic inputs from the raphe nuclei, dopaminergic inputs from the substantia nigra and ventral tegmental area, and histaminergic inputs from the hypothalamus. These systems also connect directly to each other and receive cortical feedback. Cortical signaling occurs through wired axonal connections (black arrows) and volume transmission (colored clouds), the direction and extent of volume signals reflecting ease of diffusion in different directions.

Consider the well-described ascending modulatory systems to the cortex (McCormick, 1992; Hasselmo, 1995; McCormick et al., 2020; Figure 4). These are typically presented as separate pathways with specific roles (e.g., arousal and learning). The traditional view that these systems diffusely modulate the cortex has been challenged by the presence of specific neuronal populations in each system that project to distinct cortical regions: for example, Breton-Provencher et al. (2022, p. 732) say “locus coeruleus-noradrenergic (LC-NA) activity was causal for both task execution and optimization [during learning].” But these ascending systems are connected to each other by direct lateral connections and by feedback connections from the cortex which makes it difficult to decompose and causally link them to specific functions like learning. Even if they could be activated independently by inhibiting lateral and feedback connections, volume transmission, and the modulatory tone resulting from G protein-coupled receptor activation can still generate context-dependent interactions driven by internal or external events (e.g., sensory inputs, learning, and arousal) between transmitters released at different times from different ascending systems that prevent a functional effect being causally linked to a single transmitter.

**Figure 5:**
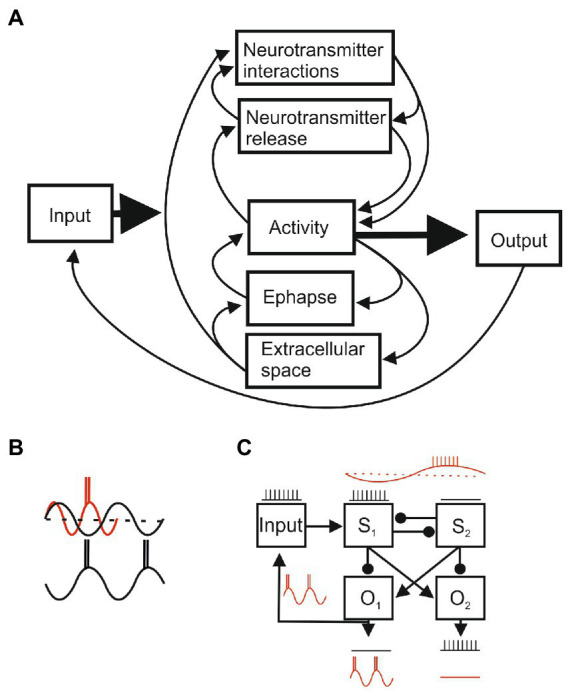
**(A)** Circular interactions between system components. Each component will have sub-systems with specific parameters that determine their states (e.g., connectivity, ion channel conductances, and synaptic strengths), initial and boundary conditions, and varying dynamic variables (e.g., membrane potential and activation or inactivation state of ion channels) that determine their outputs. The parameters of each of component can be precisely measured in reduced and quiescent preparations, but when the intact system is activated parameter values will change through inputs from other components and from external and re-afferent (feedback) inputs. Function arises from self-organized dynamic system activity that reflects the spatial arrangement of components and various temporal aspects (e.g., neurotransmitter signaling will be fast at ionotropic receptors and slower at metabotropic receptors; transmitter interactions and changes in the extracellular space will be slower still). Short and long-term plasticity can alter component properties and connections by changing initial and boundary conditions. A localized change could affect the whole system, but connection weights and temporal properties may create sub-systems where some components have a greater impact than others, and parallel pathways allow the system to function even though a pathway or component is damaged. **(B)** Oscillatory activity provides an example of temporal influences. An input may be functionally powerful (red) in the excitatory phase but ineffective in the inhibitory phase (black). In a system these effects can markedly alter outputs. **(C)** An input processed by two systems (S_1_ and S_2_) under non-oscillating conditions will evoke output 1 (0_1_) and inhibit 0_2_ (arrows reflect excitatory connections, circles inhibitory), but temporal effects during oscillations can shift the output to 0_2_. This is not a contrived situation, but reflects the common half-center organization of locomotor circuits and the influence of sensory inputs (reflex reversal; Stuart and Hultborn, 2008).

The idea that we can relate cognitive effects or behaviors to the actions of single, specific transmitters seems naïve given the evidence that multiple transmitters can interact in even simpler nervous systems. Amino acids, amines, and neuropeptides are released by successively higher rates of presynaptic activity (Verhage et al., 1991; Svensson et al., 2019). This frequency-dependence multiplexes synapses (a terminal co-localizing five transmitters could generate over 100 different combinations/signals). Nervous system activity thus alters the complement of neurotransmitters released into the extracellular space, the geometry of the extracellular space influencing the diffusion and potential for interactions along volume transmission pathways, while transmitter release and interactions will alter nervous system activity, transmitter release, the geometry of the extracellular space, and the potential for interactions, adding further circular interactions to those outlined for field effects (field effects and volume transmission are also not dissociable: transmitter-mediated or ephaptic changes in activity will alter field effects, neuronal activity, the geometry of the extracellular space, and transmitter release, diffusion, and interactions). Even without considering embodied and environmental influences, nervous system activity will reflect an equilibrium between multiple wired and non-wired circular interactions (Figure 5A) that is affected by various spatial and temporal factors (Figures 5B,C). As highlighted by McCulloch (1945), these nested circular interactions allow an equilibrium to be shifted to a new one by very small changes in activity, matching James Clerk Maxwell’s claim that life differs to physics because a “strictly infinitesimal force may determine the course of the system to any one of a finite number of equally possible paths” (see Van Strien, 2015).

While the astronomical number of cellular and synaptic components in heterarchical organizations and their degeneracy and variability offer significant practical challenges to reductionist approaches, they are in principle, if not currently in practice, achievable using reductive current approaches, albeit with the requirement that these approaches consider more than the decomposition of systems into parts. But ephaptic effects and volume transmission differ in that they not only present practical but also conceptual challenges as they reflect transient “non-wired” signals that are not reflected in anatomically defined neurons, axons, or synaptic terminals, and they require the simultaneous analysis of multiple components during ongoing activity in intact functioning systems rather than a focus on single components in the reduced quiescent or non-behaving systems often used experimentally. It could be argued that highlighting these aspects adds complexity for complexities sake and invites a pessimistic or nihilistic view of our chances of understanding. But while ephaptic signaling and volume transmission are intangible, they are not hypothetical or mysterious but are established features of nervous systems identified in reductionist analyses over several decades (Faber and Pereda, 2018; Svensson et al., 2019). It is claimed we can appeal to the decomposability of systems into component parts as a knowingly “fallible” heuristic (Bechtel, 2002; Wimsatt, 2006) or “fat-handed” approach (Romero, 2015) to gain entry to a system. There is obvious merit in simplicity, but not in being too simplistic: Occam’s razor is not that the simplest explanation is best, but that entities should not be added beyond those necessary. We may need to ask if the non-local and relational effects of ephapses and volume transmission are necessary considerations given their demonstration in multiple nervous systems and our limited ability to explain cognition and behavior based on reductionist assumptions.

## Reductionist assumptions and the basis for interventions

The philosophy of neuroscience considers the “bounds of sense… what can coherently be thought and said” (Bennett and Hacker, 2003). Considering these bounds, which include the practical and conceptual aspects of reductive approaches outlined above, is important in mechanistic schemes to avoid erroneous claims to explanation that can prevent or delay genuine advances, not least by adding the requirement to an already difficult task of identifying and undoing errors before a correct explanation can be reached. While important to explanations, considering the bounds of sense is essential for any intervention as transgressing these bounds can, and has, resulted in significant negative consequences. This section will consider approaches to intervening based on reductionist analyses and assumptions of the insight it has given us into nervous system function, using psychopathology and education as examples.

Psychoanalysis dominated early 20th century psychiatry until the mid-1950s when advances in psychopharmacology were considered to provide a more scientific basis by relating psychopathologies to abnormalities in neurotransmitter systems (e.g., the monoamine hypothesis of depression; Healy, 2015; Kendler, 2015), with interventions targeted on correcting these abnormalities. Examining biological mechanisms is as potentially useful in psychopathology as in any clinical condition providing that an explanation considers causal factors at multiple levels (see Proctor, 2012), including the aspects outlined above, and that effectiveness is established before claims for interventions are made. But this is not always the case. A spokesperson for a Huntington’s disease advocacy group expressed the dismay the group felt when a recent gene targeting trial was canceled because it worsened outcomes: “There has been so much positive noise around it; both from researchers and clinicians and from the drug company themselves. I think the community was really swept up by that hope” (see Kwon, 2021, p. 180). Similarly, claimed treatments for spinal cord injury have routinely failed (Steward et al., 2012), and the optimism that completion of the Human Genome Project will “very quickly” bring new treatments so “whole families are relieved, forever, of the curse of genetic disease” (Blakemore, 2000, p. 3) has not been realized.

An issue for mechanistic explanations and interventions in psychopathology is that despite extensive effort in trying to find them, the anatomical, cellular, or molecular features that act as diagnostic markers for neurological disorders are absent in psychiatric conditions, and diagnoses are instead made from behavior and cognition (Wang and Krystal, 2014, p. 3 wrote “there is not a single symptom of a single psychiatric disorder for which we fully understand its physiologic basis”). Absence of a biological mechanism means there is no specific biological aspect to target (e.g., normalization of an aberrant neurotransmitter level) and no end point to reach (chronic anti-depressant use and chronic depression both reduce life expectancy; Warren, 2020). Biological interventions that generate beneficial effects are still possible without a mechanistic explanation. In Parkinson’s disease, stimulation of the subthalamic nucleus can improve motor function despite current models suggesting it should worsen it (McIntyre et al., 2004). This contradiction would not matter if the stimulation reliably worked, but it is only effective in a proportion of patients, and effectiveness could presumably be improved by better understanding of the underlying neurobiological mechanisms (Piasecki and Jefferson, 2004). Penicillin provides an example: it was successfully used for some years before its mechanism was understood, but greater understanding of antibiotic mechanisms has allowed treatments to be optimized (Lobanovska and Pilla, 2017).

While interventions can be made blind to mechanisms, the history of these interventions in psychiatry is poor. In the 20th century these included ice baths, malaria-induced fevers, insulin-induced comas, electrical or drug-induced seizures, removal of teeth or parts of the digestive tract (Khazan, 2014; Davidson, 2016), and lobotomy (Breggin, 1993). These shock approaches had little or no evidence to support their use, but they were still confidently used in mainstream psychiatry: the psychiatrist R.D. Laing wrote, “I am still more frightened by the fearless power in the eyes of my fellow psychiatrists, than by the powerless fear in the eyes of their patients” (Laing, 1985, p. 18). Developments in psychopharmacology rightly removed these approaches. But although psychopharmacology assumes to target causally-relevant mechanisms, its 1950s developments reflected chance observations: the antidepressant iproniazid was developed as a treatment for tuberculosis but was coincidentally found to improve mood, its assumed inhibition of monoamine oxidases leading to the monoaminergic-hypothesis of depression; while the first antipsychotic, chlorpromazine, originated from the search for new anti-histamines, its calming effect and action on dopamine receptors promoting the dopamine-hypothesis of schizophrenia (Lehmann, 1993). Early meta-analyses suggested that psychopharmacology was the most effective approach (see Shorter, 2021), the merits of psychotherapy being questioned by claims that it did not matter what type of therapy was given, for how long, or the credentials of the therapist (see Smith and Glass, 1977). However, recent meta-analyses suggest that psychotherapy and psychopharmacology are equally effective for major depression, panic disorder, and seasonal affective disorder (Cuijpers et al., 2013; Warren, 2020; see Cipriani et al. (2018) and Munkholm et al. (2019) for discussion).

Even though we can use beneficial interventions without knowing the mechanisms underlying their effects, it is still important to consider whether relationships are causal or correlational. For example, assume that a negative life event is processed in the brain through a known cellular mechanism that lowers serotonin levels and that this in turn acts on a known mechanism that affects mood circuitry. This mechanism and the associated reduction in serotonin levels could be claimed as the cause of the depression, but at best (i.e., serotonin levels do causally influence mood) this only says how the depression occurred, not why. Knowing why is necessary to determine the optimal intervention; do we act on serotonin levels or address the negative life event? An analogy would be that hemorrhage may cause death through loss of blood volume and blood pressure leading to insufficient oxygen delivery to the brain and heart, but treatment for this would not be continual blood transfusions to maintain blood volume but treating the hemorrhage.

Even if neurobiological causation was determined, this still may not necessarily make a neurobiological intervention better than non-biological approaches (e.g., coping strategies for those with memory deficits following head injury; Tsaousides and Gordon, 2009). Phenylketonuria provides a textbook example of a causal genetic factor associated with profound psychological and neurological impairments that is successfully managed through diet, a reflection of behavior influencing lower-level effects (Rampon et al., 2000). But current views of psychopathology, as with attempts to explain normal functions, can have a neurobiological focus. For example, the perception in the autism community of a neurobiological focus in the Welcome Trust-funded Spectrum 10 K autism genetics study, led to concerns that saw the study being paused (see Sanderson, 2021). Another example comes from a Royal Society report that claimed “neuroscience provides concrete evidence of biological differences between children with ADHD and others,” despite then seemingly contradicting this by saying “There is no biological test at present” (p. 11) and that assessment is based on behavior.^2^ Pharmacological use in ADHD has increased markedly without concomitant understanding of drug mechanisms (Bachmann et al., 2017), but as with phenylketonuria there are non-biological interventions that reflect behavior in particular environments, including cognitive approaches that train children in self-evaluation (identifying issues, setting goals) and give behavioral management strategies to parents and teachers (time outs and chart/point systems; Miranda et al., 2002; Howard-Jones, 2008). These approaches require investment rather than generating profit, but the latter is not a factor that should be considered in the bounds of sense.

In addition to promoting biological explanations and approaches, constitutive and explanatory reductive views have also altered assumptions of psychopharmacology mechanisms from a drug-centered approach where drugs have some net beneficial effect on brain states underlying cognition and behavior, to a disease-centered view that sees drugs normalizing function by targeting specific biological mechanisms (e.g., excess dopamine in schizophrenia; Middleton and Moncrieff, 2019). This generates a potentially fallacious circular argument: because drugs target biological mechanisms, the mechanism is biological. Given the identification of volume transmission and neurotransmitter interactions in reductive analyses in a range of nervous systems (Svensson et al., 2019), neurobiological considerations seem to make a drug rather than disease-centered mechanism far more likely. Consider depression again: assuming that serotonin was the causal factor for depression (see Kendler, 2015) and that serotonergic drugs only affect serotoninergic systems, the effect of these drugs would not necessarily reflect a serotonin-specific effect in the brain as changes in serotonin levels along volume transmission pathways would affect numerous circular and other interactions to generate new equilibrium states (the time to establish this with global rather local physiological changes in serotonin levels may influence the delay in psychological effects despite changes in serotonin levels; Healy, 2015). Unless reasons were found to negate the need to consider volume transmission and transmitter interactions, psychopharmacological approaches won’t need drugs that more specifically target transmitter systems but knowledge of what constitutes a normal or pathological brain state, what intrinsic (e.g., personality) and extrinsic factors (social conditions) influence these states, and how (or if) we should intervene using a drug-centered approach to shift the state to one we identify as desirable.

While interventions have traditionally been poor, just as new techniques promise insight into nervous system functions new “neurotechnologies” promise better reductive interventions by using genetic engineering, stem cells, brain implants (“nanobiochips”), smart drugs (“emoticeuticals”), or downloading, “straightening out,” and re-uploading information from the brain (Geake and Cooper, 2003; Lynch, 2004; Tancredi, 2005). These claims were called the “lobotomy attitude” to reflect their limited scientific basis (Dudai, 2004), and the claims in these older references have not been realized. Proponents have made the fallacious a fortiori appeal to success in other areas, vaccination, cardiac pacemakers, control of diabetes or blood pressure, and cochlear implants (Tancredi, 2005), which offers no logical basis from which to claim success for neurotechnological interventions. The uncertainty surrounding the serotonin-hypothesis of depression and other mental disorders (Kendler, 2015) highlight that psychopathology differs to physiological conditions like diabetes where the disease-centered approach applies. Although causality is difficult to establish, understanding factors like volume transmission and transmitter and other circular interactions in heterarchic systems should provide a better basis for interventions.

Education is a recent focus for translational neuroscience. Neuroeducation claims that neuroscience can inform educational practices. This could reflect multiple approaches (Goswami, 2009), but there is again a focus by some on neurobiological mechanisms. A Royal Society report^3^ claimed that “Biological factors play an important role in accounting for differences in learning ability between individuals,” despite admitting that this conclusion is made even though “high quality information is scarce” (summary p. 5). Neuroimaging of brain areas activated in tasks like reading, speaking, writing, and counting (see Ansari and Coch, 2006) are claimed to offer insight into optimal teaching methods by facilitating specific neural mechanisms, but not what these are or how they could be targeted. The Royal Society report also says, “the brain changes constantly as a result of learning and remains ‘plastic’ throughout life” (summary p. 5). Plasticity invokes neurobiological mechanisms driven by specific inputs that alter the nervous system while emphasizing the potential influence of external or higher-level factors that drive these changes. This is highlighted in the Royal Society report which states “education is the most powerful and successful cognitive enhancer of all” (p. 1). Plasticity has been promoted as a concept that teachers can use, but plasticity just means that children learn rather than giving novel insight that would shift the emphasis from the child or school to the brain, even if we did causally understand how plasticity mechanism affect cognition (Bliss et al., 2018; Parker, 2019). Educational achievement, not a change in the brain, is the aim.

Behavioral genetics illustrates a dominant reductive neurobiological focus on cognitive abilities. This is a long-standing and contentious issue that uses heritability estimates derived from family studies of identical and non-identical twins raised together (same or different genetics in the same environment) or after adoption (different environments; Rose et al., 1985; Plomin et al., 1996) to assess the relative contribution of genetic mechanisms and the environment. These contributions are not separable and they do not have fixed values. For example, height reflects genetic and environmental influences (nutrition), but plentiful food will reduce the environmental variability and increase heritability. Various aspects complicate measures of the heritability of cognitive abilities: children alter the behavior of those around them meaning that first-born children have different environments to their siblings; adoption studies can include twins not separated at birth (allowing early environmental influences) and separation can mean one twin living with the mother and one with a relative (Rose et al., 1985). Even with complete separation at birth adoption studies usually have a restricted environmental range as adoptive parents tend to come from higher socioeconomic groups, and heritability estimates decrease when a broader socioeconomic range and thus greater environmental variability is considered (Turkheimer et al., 2003). This is mirrored in animal studies where genetically-influenced behavioral differences can disappear in enriched environments (Crabbe et al., 1999; Rampon et al., 2000).

Genetic influences on cognitive abilities are unlikely to be simple: half the genome is expressed in the brain during development and genetic effects are subject to environmental influences. External influences on cognition and behavior were thought to be limited to genetically-determined “critical periods” associated with neurogenesis and synaptogenesis, leading to claims in policy papers, the media, and brain-based education literature that neurobiological evidence suggests children should be taught before school age (Huttenlocher, 2002; McCoy et al., 2019). Nobody would deny a positive early environment is advantageous, and pre-school educational interventions are beneficial, although it is unclear what aspects are improved and for how long effects last (McCoy et al., 2019). But the claimed neurobiological mechanism needs updating: neurogenesis and synaptogenesis persist into adulthood (Lledo et al., 2006; Gould, 2007; Thompson and Wolpaw, 2014) supporting “sensitive” rather than critical periods, and pre-school interventions also reflect higher-level influences of classroom environment and teacher–child interactions (McCoy et al., 2019). If we base interventions on erroneous or simplistic mechanistic claims then beneficial effects may not occur, but a worst-case scenario is that these interventions may be deleterious. An example comes from animal studies where normally beneficial rehabilitative training given prematurely after experimentally-induced stroke can increase lesion areas and worsen functional recovery (Schallert et al., 2000).

A neurobiologically-inspired approach that has attracted significant recent interest is pharmacological cognitive enhancement in the absence of pathology. Lifestyle drugs like these can be sought even when their efficacy or safety is questioned: the withdrawn appetite suppressor fenfluramine was sought by dieters even though it caused fatal heart disease (Flower, 2004), and the ADHD drug methylphenidate is widely used as a cognitive enhancer by non-ADHD students (Koren and Korn, 2021) despite evidence that it may worsen performance (Farah et al., 2004).

Bostrom and Sandberg (2009, p. 316) appeal to reductionist neurobiological mechanisms by claiming that cognitive enhancers work by “increasing neuronal activation or by releasing neuromodulators,” a very vague mechanistic statement, but they then say that they work by “facilitating the synaptic changes that underlie learning,” and that “intervening in the permanent encoding at synapses, a process which has been greatly elucidated in recent years, [they presumably mean LTP the significance of which remains uncertain; Queenan et al., 2017; Bliss et al., 2018; Parker, 2019] is a promising target for drug development… that not only allow the brain to learn quickly, but which also facilitate selective retention of the information that has been learned” (Bostrom and Sandberg, 2009, p. 317). Very vague mechanistic claims, necessarily so given that we lack the necessary neurobiological detail, are thus turned into concrete physiological mechanisms that promise cognitive improvements by acting on memory encoding and retention.

Drugs can improve memory. Effects seem greater in poorer performers exposed to more difficult tasks, but they are modest and currently difficult to attribute to any specific biological mechanism (chewing gum can also evoke memory improvements; Wilkinson et al., 2002). A common cognitive enhancer, modafinil, directly or indirectly affects multiple transmitter systems, has varied effects on memory and other cognitive systems, and varied side-effects (Ackerman and Kanfer, 2009). Even if modafinil significantly improved real-world memory (i.e., beyond statistical effects under laboratory conditions) the bounds of sense requires asking if pharmacological interventions targeting unknown mechanisms should take priority? In addition to chewing gum, taking breaks significantly improves cognitive performance in nurses, doctors, and air traffic controllers (Smith-Coggins et al., 2006; Signal et al., 2009), a safer and more cost-effective approach. Claiming that a pharmacological cognitive enhancer is no different to using contact lenses to improve performance is a trivially false analogy^4^ (only one of these is non-invasive, readily reversible, with a known mechanism, safety, and effectiveness providing an appropriate prescription that matches the intervention to the features of the individual).

Ethical issues have been discussed extensively in cognitive enhancement, principally the unfair advantage given to those who can access and afford the drugs. But given the lack of mechanistic understanding and limited effects these discussions beg the question by assuming that significant benefits exist. But strong claims are made: Lynch (2004, p. 229) claimed that neurotechnology targeting neurobiological mechanisms will generate a “post-industrial post-informational neurosociety,” where learning and memory will be enhanced to improve competitive advantage in the workplace, sensory abilities will be improved to extend artistic expression, and emotional stability will be increased to improve personal relationships, political opinions, and cultural beliefs (what political or cultural norms are we aiming for?). Bostrom and Sandberg (2009) go further and claim cognitive enhancers could solve societal problems by making people “smarter, wiser, or more creative,” and given “the potentially enormous gains from even moderately effective general cognitive enhancements, this area deserves large-scale funding” (p. 332). In arguably, the most remarkable of the reductive “lobotomy attitude” statements they conclude by saying, “The societal benefits of effective cognitive enhancement may even turn out to be so large and unequivocal that it would be Pareto optimal to subsidize enhancement for the *poor* [my italic] just as the state now subsidizes education” (Bostrom and Sandberg, 2009, p. 334). Ignoring the ample evidence that wealth does not equal intelligence, this is some claim for drugs that lack mechanistic understanding and whose effects are mimicked by chewing gum or taking a nap.

These claims may be loosely based on the scientific approach of neurobiological reductionism, but not on science, and the bounds of sense should negate the science fiction statements and false analogies. Should we, apart from profit and convenience, appeal to pharmacological interventions with limited efficacy and unknown mechanisms (and risks?) over education that we know enhances cognition and has benefits beyond job status and salary in improving overall health and quality of life (Johnston, 2004)? Claiming that pharmacological enhancement and education are equivalent as both cause physiological changes in the brain is another false analogy (Bostrom and Sandberg, 2009): education changes the brain through the gradual integration of experiences in specific neural systems, whereas drugs instantly impose largely unknown global effects on nervous systems.

This hyperbole is balanced by Goswami (2009, p. 182), who in a paper cited only one-tenth as often as Bostrom and Sandberg (2009), considers the scientific basis and the bounds of sense of applying neuroscience to education by saying we “must proceed with caution. We cannot afford to ignore the nature of what is (and is not) possible to measure using current neuroscience techniques when framing our research questions about the brain,” and goes on to say that we should “start small, using the outcome measures that are actually possible given the current state of the art, and then to adapt educational questions to variables that we can meaningfully measure” (i.e., not try to engineer society by cognitively enhancing the poor). Bruer (2002) claims that we do not know enough about the relationship between brain physiology and learning to form meaningful links to education, yet these links are promoted. Premature neuroscience translations to education will make the classroom a laboratory. Penicillin again shows that we do not need a complete mechanism for effective interventions (Lobanovska and Pilla, 2017), but penicillin use was based on knowledge of bacterial infections and demonstrated effectiveness, a basis that pharmacological cognitive enhancements lack.

## Conclusion

Reductionist analyses that examine component parts to provide mechanistic schemes have been successful in many areas of science, including neuroscience where over several decades experimental tools have allowed increasingly precise molecular and cellular analyses and manipulations that have given insight into various aspects of nervous system function and dysfunction (e.g., the identification of biomarkers in neurology that have supplemented traditional behavioral descriptions; Anthony et al., 2014). Despite this, our success in terms of explanations or understanding of cognition and behavior and the ability to intervene has arguably been limited.

Knowledge of parts, their organization, and the functions they perform can in principle explain any system, including relational and emergent effects, providing that the necessary parts, interactions, and functions are considered. What constitutes necessary and sufficient detail remains debated (see Selverston, 1980 and the debates in the commentaries). Even if this was debate was settled in favor of a reductive approach, reductive explanations are affected by the practical difficulties of the large number of components and interactions to examine in even relatively small systems, their amenability to analysis, and limitations introduced by experimental approaches [e.g., the use of quiescent (non-behaving) and dissected or dissociated preparations]. These issues can lead to components that are less experimentally tractable being ignored for experimental convenience and functionally-relevant aspects like feedback pathways, ephapses, and volume transmission being lost. This can leave explanations based on the information available rather than the information that may be needed.

Explanations can also selectively use available information. In discussing the neuron doctrine, Gold and Stoljar (1999, p. 821) used Kandel’s sensory neuron mechanism for associative learning of the gill-withdrawal reflex in *Aplysia* as an exemplar of a psychoneural reduction, saying “we take it to be a sociological fact that Kandel’s theory is widely regarded in the neuroscientific community as the best that neuroscience can now offer in the way of explanation of behavior or the mind in fundamental neuroscientific terms.” They evaluated Kandel’s explanation at some length and concluded that it was not a successful psychoneural reduction because it still relies on psychological concepts. But the claim of a successful neurobiological reduction can be negated on far simpler grounds as it begs the question in only considering the sensory neurons and ignores known and relatively well-characterized changes in motor neurons and interneurons (see Parker, 2019). A successful neurobiological reduction would require either that the non-sensory changes were shown to be irrelevant to the explanation, or that the relative contributions of all of the effects were determined. This would require significant time and effort given the claimed forbidding complexity of the interneuronal connections (Hawkins et al., 1981), but aspects should not be ignored for convenience.

Highlighting the limitations and challenges of reductive analyses should not be taken as support for the opposing view that lower-level detail is irrelevant and we should instead focus on higher-level computations and representations (Silberstein and Chemero, 2013; Barack and Krakauer, 2021). The latter offer descriptions of population effects that reductive do not usually provide, but they also offer limited explanations (only approximately 30% of the variance in visual cortex responses to natural stimuli can be accounted for by current computational coding models; Bertalmio et al., 2020). One obvious benefit of reductive analyses is to provide detail that can inform and constrain higher-level abstract or phenomenological models. Hodgkin and Huxley (1952, p. 541) cautioned their action potential model, “must not be taken as evidence that our equations are anything more than an empirical description…An equally satisfactory description of the voltage clamp data could no doubt have been achieved with equations of very different form”: their model was ultimately supported by molecular analyses of channel properties over three decades later (Catacuzzeno and Franciolini, 2022).

Dichotomies like that between reductionist and representational approaches have stymied various fields (e.g., sensory vs. centrally driven locomotion, and presynaptic vs. postsynaptic expression of LTP; Stuart and Hultborn, 2008; Lømo, 2018). The need to consider effects at multiple levels has been raised repeatedly. Bernard (1927) wrote, “Admitting that vital phenomena rest upon physico-chemical activities, which is the truth, the essence of the problem is not thereby cleared up…when we wish to ascribe to a physiological quality its value and true significance, we must always refer to this whole.” Sherrington made a similar claim: although he recognized the importance of relational interactions in nervous systems in calling reflexes a “convenient fiction,” he highlighted the benefits of a reductive approach in saying “it is helpful in analyzing complex reflexes to separate from them components which we may consider apart and therefore treat as though they were simple reflexes” (Sherrington, 1906, p8). From this reductive approach, he provided functional evidence for synapses and rules of synaptic integration still relevant today. Bullock also followed a reductionist approach in his neuroethological analyses (see Zupanc and Zupanc, 2008): he was the first to examine synaptic transmission using paired recordings in the squid and identified electrical synapses in the crustacean cardiac ganglion. But he also examined sensory and motor principles at behavioral levels, using a neuroethological focus on the species-dependent differences that reflected adaptations to ecological and behavioral requirements. Bullock criticized the “mutual disparagement” between single neuron and population approaches, saying “Each of these approaches is a window and a quite inadequate one. We need both and the combination of the two and still others to untangle this most complex of known systems” (Bullock, 1995, p. 231).

In addition to being counterproductive, debate, or for Bullock the mutual disparagement, over the relative merits of representational/computational and reductionist approaches seems premature given the lack of necessary detail and clarity of definitions. For example, definitions of representations vary (Barack and Krakauer, 2021) and numerous abstract computational terms and analytical approaches are used that have only tangential links to each other and to neurobiology (Silberstein and Chemero, 2013). Neurobiological details need to be considered to prevent computational aspects becoming “descriptive conveniences” (Warren, 2012). Bennett and Hacker (2003, p. 147) wrote, “To say that the mind has ‘access’ to the ‘internal representation’ produced by the brain is no less mysterious than the Cartesian claim that the mind has access to an image on the pineal gland.” Does it matter that a synapse is a complicated molecular system of multiple protein–protein interactions (Wilhelm et al., 2014) rather than a number in a matrix: it probably does. Conversely, claims of mechanistic explanations of cognitive functions and behaviors from neurobiological analyses seem premature as they are predicated on data that fails to satisfy the minimal neurobiological criteria for understanding (e.g., Selverston, 1980), criteria that need to be updated and expanded to include variable relational effects in heterarchical systems, ephaptic fields, volume transmission, and transmitter interactions.

Claiming that cognitive explanations need to account for state spaces across many spatio-temporal scales (e.g., Churchland and Churchland, 1990; Barack and Krakauer, 2021) repeats Lashley’s dilemma (see above; Lashley, 1942). Whether the non-local relational aspects discussed here could help link representations and state spaces across different spatio-temporal scales, as Bullock (1959) suggested, remains an open question given the limited consideration of these phenomenon. Ephapses will provide spatially and temporally varying activity in neuronal populations, while volume transmission and transmitter interactions will allow spatially and temporally varying context-dependent effects driven by changes in internal or external conditions (e.g., sensory or cortical activity evoking modulator release from brainstem modulatory systems). These effects should also be considered by those who claim functions “bottom-out” in genes, molecules, neurotransmitters and neurons. Kaplan and Craver (2011, p. 603) write, “we oppose strong dynamicist and functionalist views according to which mathematical and computational models can explain a phenomenon without embracing commitments about the causal mechanisms,” but the same applies to mechanistic views that fail to embrace known mechanisms that alter simple mechanistic views and complicate causal claims.

Placing representational or computational aspects in neurobiological terms is not impossible: a visual receptive field is a representation of external space that can be reduced, although not yet completely, to the connectivity of retinal neurons; analyses of synaptic information transfer consider representational aspects in neurobiological terms (Laughlin et al., 1998), and graph theoretical approaches group neurons into functional assemblies or motifs (Hadjiabadi and Soltesz, 2022). While the latter are presented as novel insights, these motifs have been considered in neurobiology for many years albeit under the original term of building-blocks (Getting, 1989). Despite claims that the identification of an anatomical motif can predict function (Morgan and Lichtman, 2013), we know from reductive analyses that this is not possible from identification of a motif alone: Elson et al. (2002) showed that a single two-neuron motif can generate alternating or synchronous activity depending on the functional properties of their connections. But by combining computational approaches with connectomic data and imaging cell populations at single cell resolution (e.g., zebrafish or hippocampal slices) links are now being made between single cell and population effects (see Hadjiabadi and Soltesz, 2022).

Linking lower and higher-level effects nevertheless remains the major open question in neuroscience. Claims to Kuhnian paradigm shifts and scientific revolutions (Kuhn, 1962), which are generally rare events, are frequently made in neuroscience (Parker, 2018). These claims could, in principle reflect genuine revolutionary advances; a reflection of the pre-paradigm state as neuroscience tries to find its optimal approach from among the various reductionist or representational approaches suggested; or evidence of a field in a scientific crisis as claimed or promised explanations and interventions have failed to materialize (Parker, 2019). A scientific revolution does not occur when current views face anomalies (*cf* Barack and Krakauer, 2021), anomalies can instead entrench views, but when an alternative approach is offered that overcomes the addresses the issues that have held a field back. Attention focused on the relational aspects originally highlighted by Lashley (1942), McCulloch (1945), and Bullock (1959) may provide insight that suggests alternatives to current paradigms and dichotomies that move the field forward.

## Author contributions

The author confirms being the sole contributor of this work and has approved it for publication.

## Conflict of interest

The author declares that the research was conducted in the absence of any commercial or financial relationships that could be construed as a potential conflict of interest.

## Publisher’s note

All claims expressed in this article are solely those of the authors and do not necessarily represent those of their affiliated organizations, or those of the publisher, the editors and the reviewers. Any product that may be evaluated in this article, or claim that may be made by its manufacturer, is not guaranteed or endorsed by the publisher.

## Footnotes

^1^ https://braininitiative.nih.gov/

^2^ https://royalsociety.org/-/media/Royal_Society_Content/policy/publications/2011/4294975733.pdf

^3^ https://royalsociety.org/-/media/Royal_Society_Content/policy/publications/2011/4294975733.pdf

^4^ https://www.theguardian.com/society/2007/nov/08/health.lifeandhealth
